# Radiation safety behavior model for the radiological science departments of Universities in Korea

**DOI:** 10.12669/pjms.344.14874

**Published:** 2018

**Authors:** Pyong Kon Cho, Yong Min Kim, Hyon Chol Jang, Yeo Ryeong Jeon, Eun Ok Han

**Affiliations:** 1 Dr. Pyong Kon Cho, Department of Radiological Science, Daegu Catholic University, Gyeongsan, Korea; 2 Dr. Yong Min Kim, Department of Radiological Science, Daegu Catholic University, Gyeongsan, Korea; 3 Dr. Hyon Chol Jang, Department of Radiological Technology, Suseong College, Daegu, Korea; 4 Ma. Yeo Ryeong Jeon, Department of Radiological Science, Daegu Catholic University, Gyeongsan, Korea; 5 Dr. Eun Ok Han, Department of Education & Research, Korea Academy of Nuclear Safety, Seoul, Korea

**Keywords:** Haddon matrix, Radiation protection, Risk education, Safety training, Social cognitive theory

## Abstract

**Objectives:**

This study was to employ the major variables relating to radiation safety that were derived using the Haddon Matrix to develop a radiation safety behavior model based on social cognitive theory that can be applied to improve the radiation safety behaviors of professors and students in radiological science departments.

**Methods:**

The safety levels of students and professor in radiological science departments were analyzed in order to design a radiation safety behavior management model based on the Haddon Matrix and social cognitive theory, which can be used to enhance radiation safety strategically. The survey was administered on April 23, 2015, to professor and students of 45 universities around South Korea with established radiological science departments, and the investigation lasted for 30 days.

**Results:**

When multiple linear regression analysis was conducted taking the radiation safety behaviors of the students in the radiological science departments as the dependent variables, it was found that these behaviors were affected by self-efficacy, knowledge of the materials, and attitudes towards the materials, in order of greatest to least influence.

**Conclusion:**

Since the general view on radiation safety management in universities is also changing to a maximalist attitude, the level of safety management must be increased accordingly.

## INTRODUCTION

As of 2015, radiological science departments in 45 universities around South Korea use radiographic equipment to offer practical training to students. The diagnostic radiographic instruments used in these departments include equipment for projection radiography, fluoroscopy, mammography, and computed tomography. The current regulatory agency manages these educational institutions by reviewing the safety of their radiation source designs and by inspecting and evaluating safety-related issues regarding the operating facility from its design phase to its practical use. Even for identical radiation-generating devices, different legal provisions are applied among medical, veterinary, and educational institutions. Although various radiation system safety analysis methods are employed and institution-specific radiation safety guidelines are in place^1^, such efforts are insufficient in terms of preventing radiation accidents by taking the appropriate precautionary measures due to the lack of funding for radiation safety management, as well as the lack of practical support by South Korean universities using radiation-generating devices.

Furthermore, the unrealistic nature of these radiation safety management procedures and regulations often causes problems for universities in their radiation protection efforts.^2^ Therefore, Lim (2010) addressed the need for systematic radiation risk education, since radiation exposure knowledge and awareness of risk prevention methods were found to be insufficient.^3^ Similarly, Moon (2015) reported that students majoring in radiology lacked awareness of safe exposure doses, despite having taken radiation safety courses, suggesting the need for a new and more specialized safety education program.^4^ Moreover, it is necessary for experts on the various effects of radiation use as well as radiation educators to consider safety from a precautionary perspective.^5^

In particular, health and medical service personnel must maintain proper understanding and awareness to prevent the radiation exposure of patients and their families^6^, and students majoring in radiological sciences and proceeding into health and medical professions must undergo a systematic educational program to enhance their knowledge and to promote appropriate attitudes and behaviors regarding radiation safety. Accordingly, the objective of this study was to employ the major variables relating to radiation safety that were derived using the Haddon Matrix to develop a radiation safety behavior model based on social cognitive theory that can be applied to improve the radiation safety behaviors of professors and students in radiological science departments.

## METHODS

In this research, previous studies and radiation safety management regulations in universities were analyzed, variables necessary for radiation safety management were determined via field research, the actions necessary for radiation safety management were deduced by weighting the major safety-related variables based on consultations with three experts, and survey questions were developed subsequently. The final survey was modified and supplemented by conducting two preliminary surveys. The survey was administered on April 23, 2015, to professor and students of 45 universities around South Korea with established radiological science departments, and the investigation lasted for 30 days. The survey participants included 49 professor—40 males (81.6%) and nine females (18.49%)—and 840 students—446 males (53.1%) and 394 females (46.9%)—in radiological science departments.

Four preventative safety management variables were identified using the Haddon Matrix, namely, a human factor, a material (i.e., radiation-generating device or radiation source) factor, a social environment factor, and a physical environment factor and were subsequently employed to design. Based on each participant’s knowledge, attitude, and behavior, as elucidated through these questions, the values of the variables in each of the four categories were determined for each participant. Furthermore, four self-efficacy questions and four questions related to expectations that affect human behavior were asked.

Cronbach’s alpha values for survey reliability were acquired for the professor members’ behaviors (material factors 0.851, human factors 0.890, social environment factors 0.895, and physical environment factors 0.822), attitudes (material factors 0.8909, human factors 0.8954, social environment factors 0.902, and physical environment factors 0.944), safety management expectations (0.722), and self-efficacies (0.574). These values were also obtained for the students’ behaviors (material factors 0.916, human factors 0.875, social environment factors 0.875, and physical environment factors 0.869), attitudes (material factors 0.930, human factors 0.935, social environment factors 0.911, and physical environment factors 0.944), safety management expectations (0.894), and self-efficacies (0.546). The Haddon Matrix employed in this study was developed in 1968 and provides a plausible framework for identifying various countermeasures against problems that arise from understanding the factors that cause injuries.^7^,^8^ In this study, the Haddon Matrix was employed to identify the factors leading to problems in radiation safety behavior, and to enable the systematic design of countermeasures against these factors. Social cognitive theory stipulates that an individual’s behavior and cognition affects his or her behavior in the future, and that behavior, personal factors, and environmental factors are related to one another during the learning process. Therefore, social cognitive theory, which emphasizes reciprocal determinism, was incorporated into this study as a main component in determining human behavior.

### Statistical Analysis

SPSS 15.0 and AMOS 7.0 were utilized to derive the mean, standard deviation, and path analyses. In order to evaluate the model fit, that is, whether the model was appropriate for the given data, the chi-squared statistic, mean-squared statistic, number of degrees of freedom, Goodness-of-Fit Index (GFI), Adjusted Goodness-of-Fit Index (AGFI), Root-Mean-Square Error of Approximation (RMSEA), Comparative Fit Index (CFI), and Tucker–Lewis Index (TLI) were used.

## RESULTS

### Level of radiation safety according to the Haddon matrix

For the professor, all of the variables related to radiation safety had values over 3 (out of 5), so they were slightly above average. For the students, one variable related to radiation safety scored a low value of 2 (out of 5). The values of the variables corresponding to medical examination before initial practical training, grounding equipment inspections, electric current, regularity of radiation safety committee meetings, and notification of expected maximum exposure dose were comparatively low, there is room for improvement in all four of the categories that were identified using the Haddon Matrix. Regular maintenance of X-ray instruments and appropriate use of radiation protection equipment are required as part of the safety management of diagnostic radiographic instruments, and it is necessary to reduce the time that the person performing maintenance is exposed to radiation or for him or her to maintain a safe distance from the equipment in order to reduce his or her X-ray exposure. However, the results indicate that more stringent regulations are necessary in this regard to ensure a sufficient level of safety^9^,^10^ as seen in Table-I.

**Table-I:** Level of radiation safety according to the Haddon matrix.

Human Factor			Source Factor			Environment Factor					

						Social Environment			Physical Environment		

Type	Professor	Student	Type	Professor	Student	Type	Professor	Student	Type	Professor	Student
Regular Measurement of Radiation Dose	4.06±0.99	3.33±0.72	Index for the Management of Radiation Generating Devices (Checklist)	4.08±0.91	3.65±0.33	Safety Communication Between Student, Professor and Radiation Safety Manager	4.24±0.83	3.75±0.99	Locking Device for Laboratories	4.78±0.51	4.04±0.75
Medical Examination Before Initial Practical Training	3.86±1.43	2.82±0.72				Testing System for Radiation Exposure Dose, Expected Exposure Dose, and Examination Results	3.86±1.10	3.31±0.73	In—Use Notification on Doorways	4.47±1.02	3.88±0.79
Use of Personal Dosimeter	3.94±1.39	2.75±0.75	Quality Control on Irradiation Dose (Tube Voltage, Tube Current, Irradiation Time)	4.12±1.03	3.61±0.32	Influence of Radiation Safety Manager	4.04±1.00	3.51±0.74	Interlocking Device for Opening and Closing the Operating Facility Doorways	3.88±1.39	3.49±0.73
Practice of Radiation Shielding	4.53±0.87	3.86±0.74				Allocation of Time for the Education of Radiation Safety Management Regulations	3.84±1.20	3.61±0.75			
Reduction in Exposure Time	4.71±0.68	4.00±0.91	Performance of Radiation Field Control Device	4.08±0.93	3.63±0.50	Awareness on the Importance of Radiation Safety Management for Managers	3.82±1.15	-	Notification of Radiation Protection Warning and Maximum Expected Exposure Dose	3.73±1.15	3.49±0.73
Distance Maintenance from Radiation Source	4.63±0.73	3.99±0.86							Availability of Protective Equipment (Lead Apron, Lead Scarf, Lead Glasses, Etc.)	4.43±0.91	3.97±0.73
Radiation Protection Training	4.37±0.95	3.96±0.72	Storage and Use of Radiation Generating Device in Radiation Controlled Area	4.63±0.73	3.96±0.71	Registration as Radiation Workers by Professors and Instructors	4.49±0.92	-	Notification of Contact for Radiation Safety Manager	4.55±0.91	3.69±0.80
Practical Training in the Radiography of the Human Body	4.49±0.87	3.44±0.72				Regular Meeting of Radiation Safety Committee	3.47±1.16	-			
Familiarity with the Precautions for Protection Against Radiation Hazard	4.55±0.77	3.83±0.73	Inspection of the Half-Value-Layer, External Leakage Current, and Grounding Equipment	3.55±1.14	3.46±0.71	Delivery of Radiation Safety Management By a Regulatory Agency	3.86±1.02	-	Installation of Radiation Measuring Device	4.37±0.76	3.57±0.75
Familiarity with the Escape Route in case of Emergency	4.18±0.99	3.62±0.72							Availability of Alderson RANDO Phantom	4.69±0.77	4.03±0.71

* The value of each factor was measured out of 5 and was represented by the mean and standard deviation (m ± sd). Knowledge, attitude, and behavior were rated from a minimum of 1 to a maximum of 5, whereas expectation and self-efficacy were rated from a minimum of 1 to a maximum of 7.

* The professor in the radiological science departments had a knowledge level of 4.87±0.31, an attitude level of 4.67±0.43, and a behavior level of 4.20±0.64. Their level of expectation about radiation safety management was 6.09±0.82, whereas their self-efficacy was 6.05±0.73.

* The students in the radiological science departments had a knowledge level of 4.20±0.97, an attitude level of 4.31±0.60, and a behavior level of 3.64±0.74. Their level of expectation about radiation safety management was 5.46±1.13, whereas their self-efficacy was 4.74±0.99.

### Radiation safety behavior model

**Fig.1:**
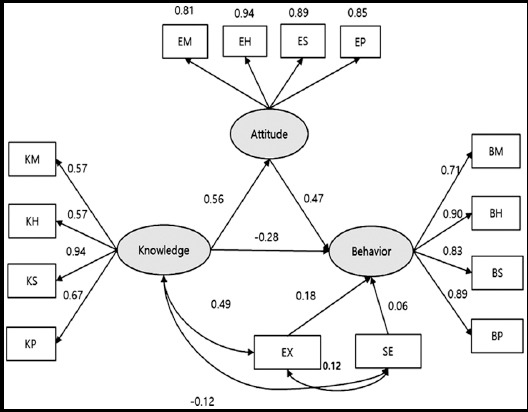
Professor Radiation Safety Behavior Model Generated Using Structural Equation Modeling. *BM (behavior of material factors), BH (behavior of human factors), BS (behavior of social environmental factors), BP (behavior of physical environmental factors), KM (knowledge of material factors), KH (knowledge of human factors), KS (knowledge of social environmental factors), KP (knowledge of physical environmental factors), EM (attitude of material factors), EH (attitude of human factors), ES (attitude of social environmental factors), EP (attitude of physical environmental factors), EX (Expectation for behavior), and SE (Self-Efficacy).

**Fig.2:**
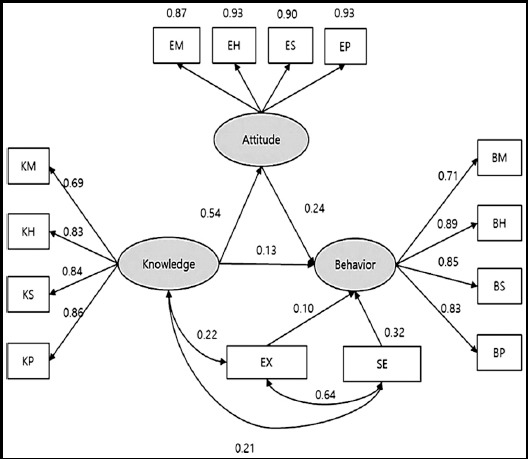
Student radiation safety behavior model generated using structural equation modeling.

The radiation safety model for the professor in the radiological science departments was deemed to be an acceptable fit based on the RMSR, whereas the RMSEA, GFI, NFI, and AGFI indicated an unacceptable fit as seen in Table II and III and Fig.1. In contrast, the radiation safety model for the students in the radiological science departments was found to be an acceptable fit based on the RMSR, RMSEA, GFI, and NFI, while the AGFI indicated that the fit was close to acceptable as seen in Table II and III and Fig.2.

**Table-II:** Evaluation of fit of radiation safety behavior model.

Type	RMR	RMSEA	GFI	AGFI	NFI	x^2^	Df	P
Professor	0.069	0.153	0.720	0.586	0.717	149.434	71	0.000
Student	0.069	0.079	0.930	0.896	0.949	427.292	71	.000

*The goodness of fit for the model and sample data were verified using the chi-square (χ^2^) statistic (p > 0.05 is desirable). Because the χ^2^ statistic is sensitive to the sample size, if the sample size is about 200 or bigger, the result is presented as if there were a difference, even though there is no statistically significant difference. In addition, if the sample size is 100 or smaller, the result is presented as if there were no difference, even though there is a statistically significant difference. Accordingly, (A)GFI, RMSEA, RMSR, and NFI are most widely used for performing goodness-of-fit evaluations of structural function models

**Table-III:** Path analysis of radiation safety behavior.

	Regression Weights			β	B	S.E.	C.R.
Students	Attitude	⇐	Knowledge	0.560	1.687	0.575	2.934^*^
	Behavior	⇐	Expectation	0.182	0.118	0.109	1.076
	Behavior	⇐	Self-Efficacy	0.063	0.047	0.108	0.439
	Behavior	⇐	Attitude	0.469	0.599	0.255	2.352^*^
	Behavior	⇐	Knowledge	-0.280	-1.078	0.849	-1.269
	Expectation	⇔	Self-Efficacy	0.125	0.072	0.084	0.849
	Knowledge	⇔	Self-Efficacy	-0.116	-0.011	0.015	-0.742
	Knowledge	⇔	Expectation	0.487	0.054	0.023	2.414^*^

	**Regression Weights**			**β**	**B**	**S.E.**	**C.R.**

Students	Attitude	⇐	Knowledge	0.540	0.340	0.025	13.456^**^
	Behavior	⇐	Expectation	0.095	0.058	0.025	2.286^*^
	Behavior	⇐	Self-Efficacy	0.321	0.225	0.03	7.48^**^
	Behavior	⇐	Attitude	0.242	0.304	0.051	5.947^**^
	Behavior	⇐	Knowledge	0.132	0.104	0.033	3.125^**^
	Expectation	⇔	Self-Efficacy	0.644	0.726	0.047	15.397^**^
	Knowledge	⇔	Self-Efficacy	0.209	0.183	0.034	5.42^**^
	Knowledge	⇔	Expectation	0.219	0.219	0.039	5.637^**^

Knowledge, attitude, and behavior are major variables in traditional education models. Hazardous materials (radiation-generating devices), human factors, social environment, and physical environment are major variables in the Haddon Matrix.

## DISCUSSION

In the 1980s, the sudden increase in the use of social cognitive theory to analyze behavior led to ongoing research on self-efficacy and expectations, which has resulted in learners now being regarded as active, rather than passive, beings.^11^,^12^ The importance of self-efficacy and expectations in determining radiation safety behavior is demonstrated by this study’s results as well. Self-efficacy is the judgment of one’s own ability to structure and perform an action in order to accomplish a goal. Therefore, the degree of confidence in one’s own ability is equivalent to one’s self-efficacy in performing an action that requires that ability; this self-efficacy is expressed through behavior.^13^ An individual’s attitude indicates his or her degree of positive or negative perception of performing a specific action^14^, as well as his or her conviction when performing that action. Attitude has long been accepted in social psychology as a factor that predicts behavior.^15^ Behavioral scientists have begun to apply social cognitive theory creatively to advance educational processes and techniques based on cognitive variables, which can be employed to enhance the possibility of behavioral change.^16^-^19^ Since radiation exposure during medical treatment can affect the medical professor as well as the individuals looking after the patients, radiation specialists must be properly educated at universities in order to enhance their radiation safety behaviors. Appropriate training would produce health and medical service personnel who would practice effective radiation management methods in their workplaces.^20^

## CONCLUSIONS

Enhancing radiation safety behavior necessitates not only emphasis on knowledge and appropriate attitudes in education, but also the instillation of expectations about the outcomes of radiation safety management, as well as the implementation of a personality development program that prioritizes increasing self-efficacy over imparting specialized knowledge.
